# Supplementary material for: Transcriptional Analysis of lncRNA and Target Genes Induced by Influenza A Virus Infection in MDCK Cells
Source: Vaccines (Basel). 2023 Oct 14;11(10):1593. doi: 10.3390/vaccines11101593 (PMC10610897; doi:10.3390/vaccines11101593)
Supplement: Supplementary file 1 [file vaccines-11-01593-s001.zip › Supplementary Figure S1.Full-length blotsgels.pdf]

Full-length blots

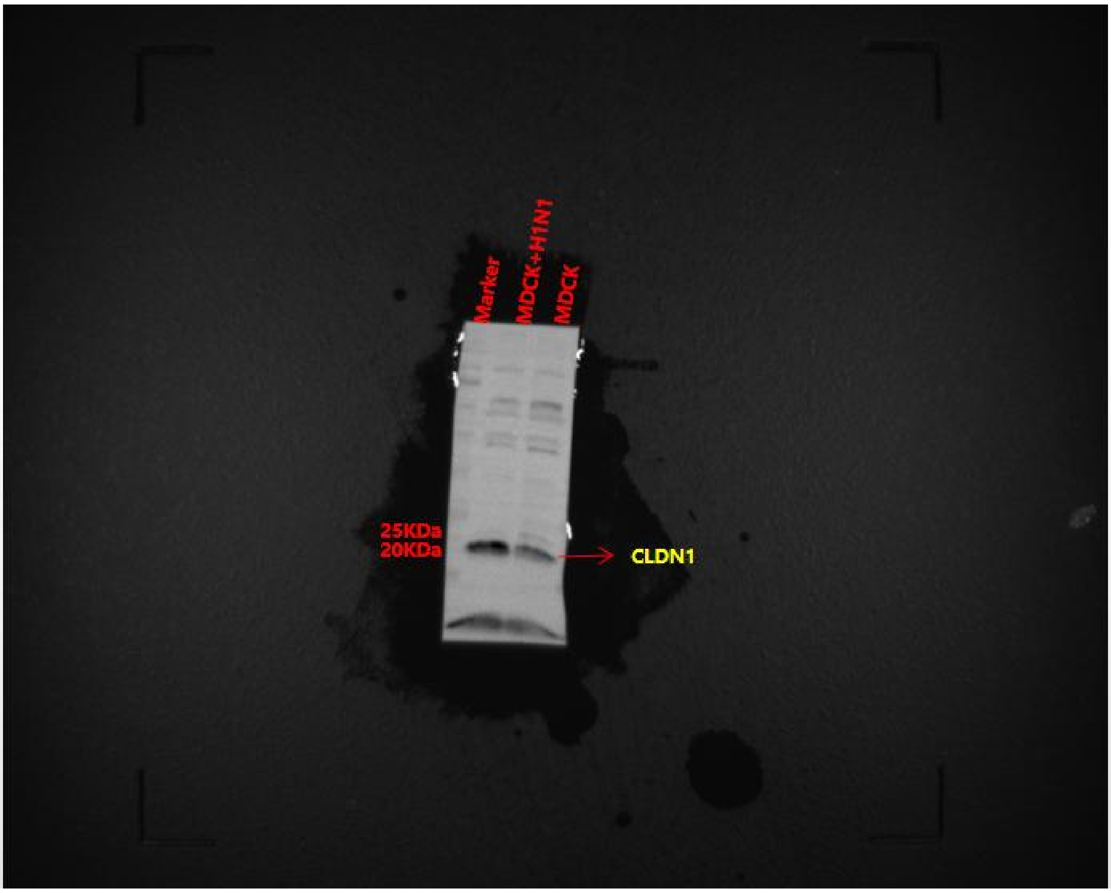

A. Verification of CLDN1 (22KDa) full-length blots differential protein expression in IAV H1N1-infected MDCK cells.

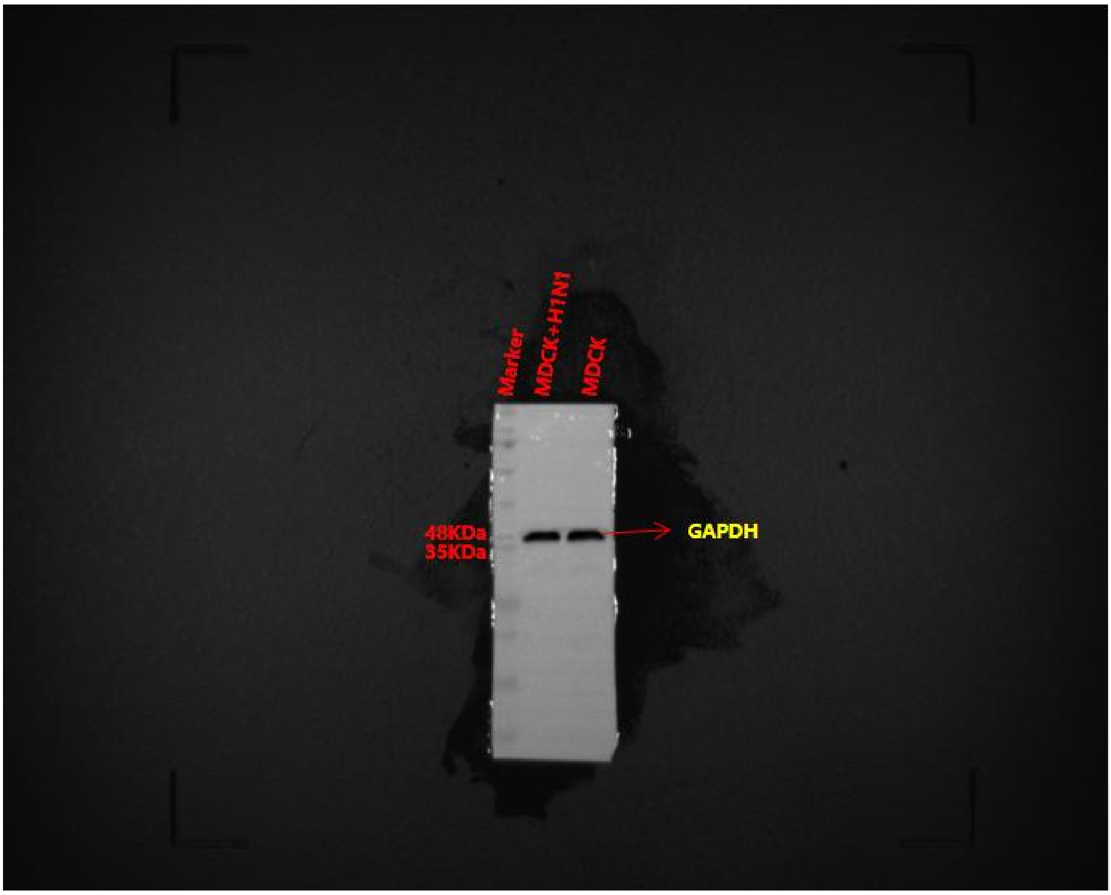

B. GAPDH (36KDa) full-length blots .

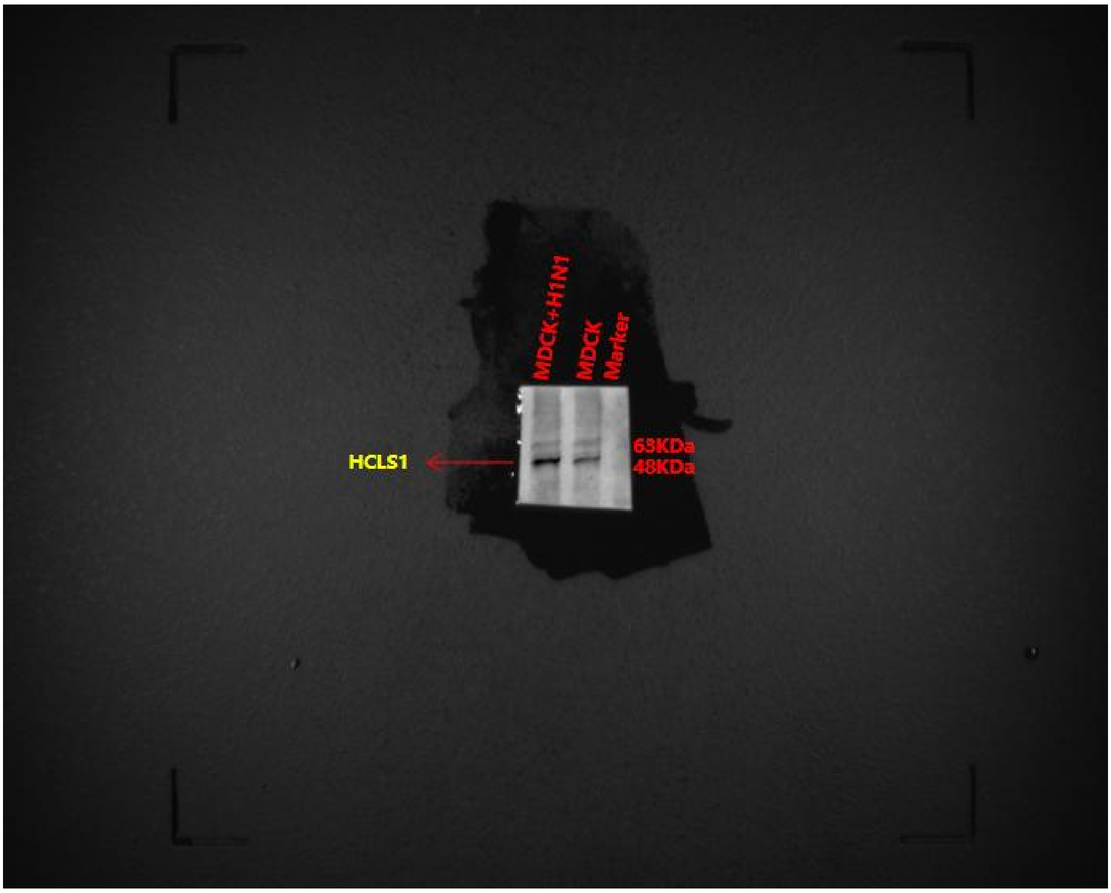

C. Verification of HCLS1 (54KDa) full-length blots differential protein expression in IAV H1N1-infected MDCK cells.

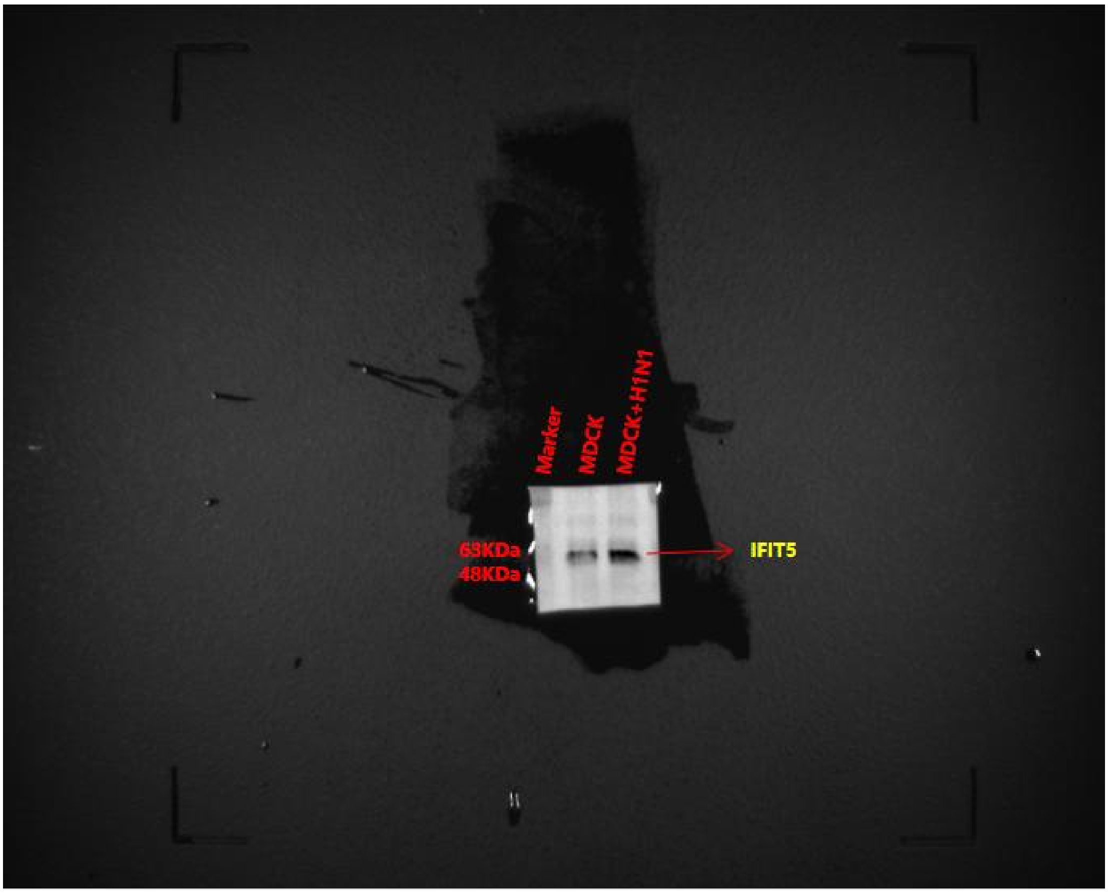

D. Verification of IFIT5 (58KDa) full-length blots differential protein expression in IAV H1N1-infected MDCK cells.

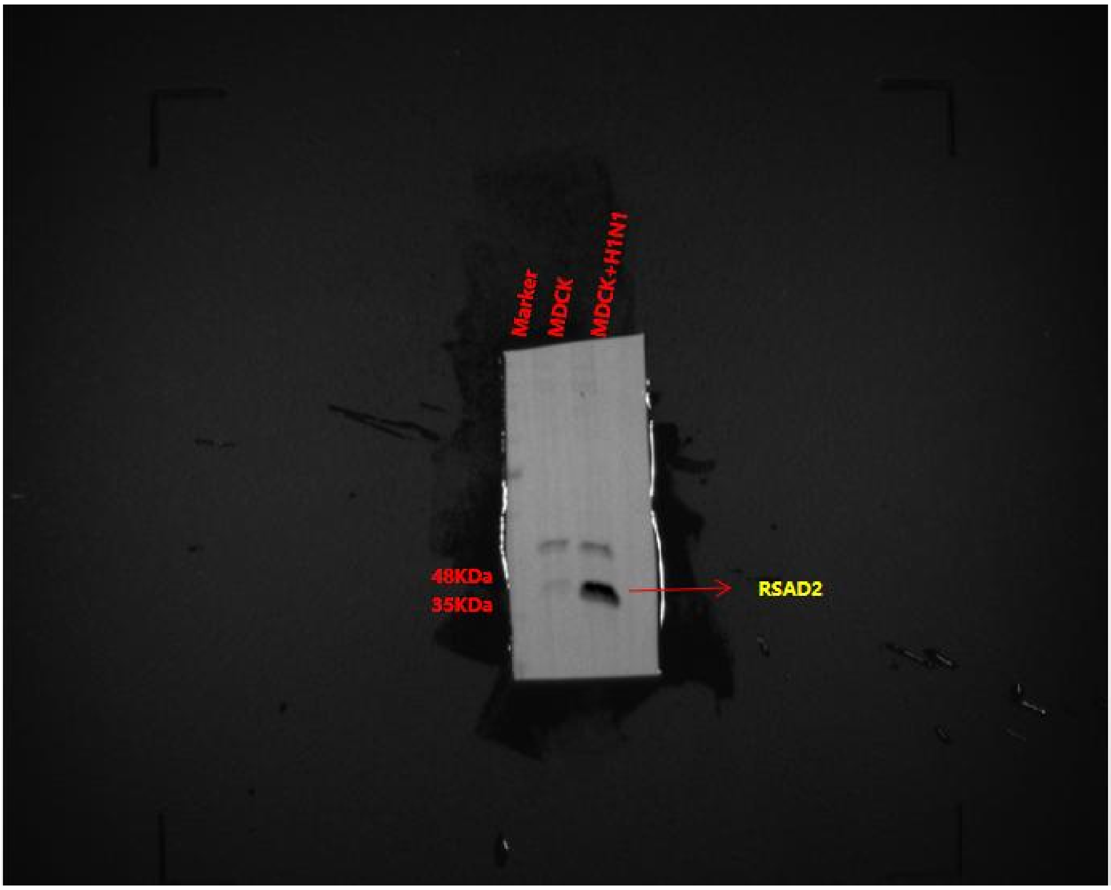

E. Verification of RSAD2 (43KDa) full-length blots differential protein expression in IAV H1N1-infected MDCK cells.
